# Treatment of Post-stroke Dysphagia With Interferential Current: Three Case Reports and a Review of the Literature

**DOI:** 10.7759/cureus.54806

**Published:** 2024-02-24

**Authors:** Bülent Alyanak, Fatih Bağcıer, Serkan Kablanoğlu

**Affiliations:** 1 Physical Medicine and Rehabilitation, Kocaeli University, İzmit, TUR; 2 Physical Medicine and Rehabilitation, Başakşehir Çam ve Sakura City Hospital, İstanbul, TUR

**Keywords:** dysphagia, interferential current, stimulation, stroke, swallow, therapy

## Abstract

Stroke is damage to the central nervous system due to vascular pathology. Stroke causes many complications. One of the most important of these complications is dysphagia. Dysphagia is a major cause of morbidity and mortality. In recent years, the benefits of using interferential current (IFC) stimulation in the treatment of dysphagia due to various etiologies have been demonstrated. However, there are significant gaps in the literature regarding patient populations, treatment procedures, and evaluation of treatment response. Here, we report the treatment of three cases of dysphagia after ischemic stroke with IFC stimulation and review the current literature. The patients had no previous treatment for dysphagia and were using only compensatory methods. This case report highlights the benefit of IFC stimulation in the treatment of post-stroke dysphagia both clinically and videofluoroscopically. It should be kept in mind that IFC stimulation may be an important alternative in the treatment of post-stroke dysphagia.

## Introduction

Stroke is the second leading cause of death and disability worldwide [1]. One of the major complications of stroke is dysphagia. Dysphagia occurs in approximately 20-70% of stroke patients in the acute phase, 10-40% in the subacute phase, and 5-25% in the chronic phase [2]. Although swallowing is analyzed in different stages, the process is complex and should be evaluated as a whole. The central coordination of this complex sensorimotor task involves an extensive network of cortical, subcortical, and brainstem structures [3]. Dysphagia can lead to complications, such as dehydration, malnutrition, weight loss, sarcopenia, increased risk of infection, aspiration, and aspiration-related pneumonia [2]. Therefore, early diagnosis and intervention of dysphagia after stroke are important. There are many treatment methods, such as postural changes, changing the viscosity of food, oropharyngeal exercises, swallowing maneuvers, thermal tactile stimulation, and transcutaneous electrical stimulation, which are used in swallowing rehabilitation for patients with dysphagia [4-6].

Transcutaneous neuromuscular electrical stimulation was approved by the Food and Drug Administration (FDA) in 2001 for the treatment of dysphagia [7]. It is used to stimulate muscle contractions and/or activate sensory pathways through "motor" and "sensory" stimulation [8,9]. Motor stimulation aims to induce muscle contraction, increase muscle strength, and improve laryngeal elevation by efferent stimulation of the pharyngeal muscles. Sensory stimulation can increase sensory input to the swallowing center of the brainstem by stimulating afferent nerves. Periodic sensory stimulation may reorganize the motor cortex and induce cortical neuroplasticity that facilitates swallowing. This may improve the swallowing reflex and prevent aspiration [10,11]. Therefore, afferent nerve stimulation may be a key factor in dysphagia rehabilitation, as opposed to efferent nerve stimulation, which induces muscle contraction and improves swallowing [12]. For this purpose, interferential current (IFC) stimulation at the sensory threshold stimulates afferent nerves without inducing muscle contractions.

In recent years, articles have been published investigating the efficacy of IFC stimulation in dysphagia [13-17]. Although there are studies evaluating the efficacy of IFC stimulation in dysphagia of various etiologies, studies evaluating the efficacy of IFC stimulation in post-stroke dysphagia using videofluoroscopic parameters are limited and very few. Therefore, this study evaluated the efficacy of IFC stimulation treatment in three patients with post-stroke dysphagia using videofluoroscopy, the gold standard in swallowing evaluation. This article discusses three cases of post-stroke dysphagia demonstrating the efficacy of IFC stimulation in light of the literature.

## Case presentation

All patients underwent a clinical examination for swallowing both before and after treatment and were assessed using the functional oral intake scale (FOIS) [18] and the videofluoroscopic swallowing study (VFSS) [19]. Lateral plane images were obtained on the VFSS with the fluoroscopic tube focused anteriorly on the lips, posteriorly on the pharyngeal wall, superiorly on the hard palate, and inferiorly on the seventh cervical vertebra for the lateral plane. The contrast agent was iohexol mixed 1:1 with liquids and semi-solids. Liquids and semi-solids were administered in 5 ml and solids were administered in the form of a biscuit and images were obtained. Solids were given in the form of biscuits coated with contrast medium just before the examination to prevent softening. The penetration-aspiration scale (PAS) [20] and the Eisenhuber residue scale (ERS) [21] were scored using the VFSS. Three swallowing trials were made. The worst scores from the swallowing trials were used. All patients underwent postural maneuvers and food consistency modification as compensatory methods and IFC stimulation therapy.

**Figure 1:**
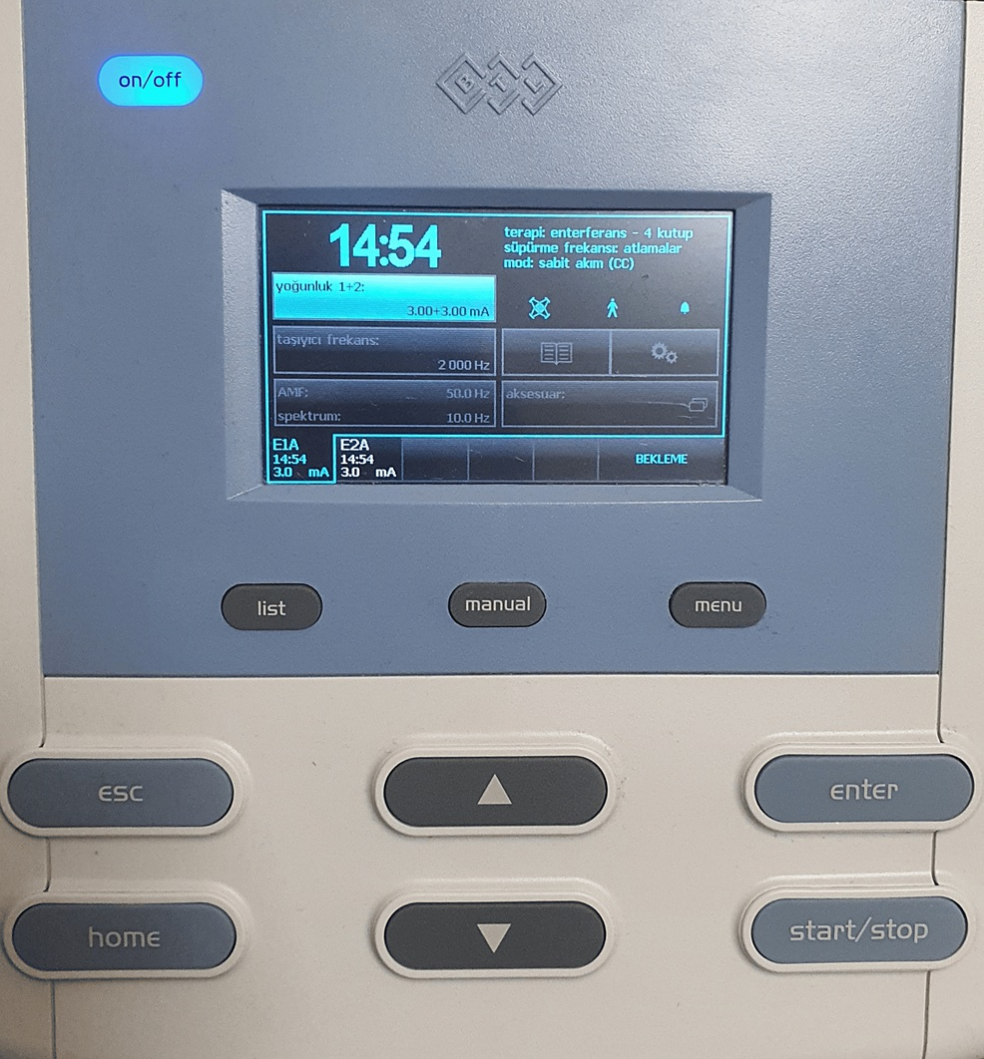
Current parameters of the device. The current density of both outputs was set to 3 mA. The carrier frequency was set to 2,000 Hz, amplitude modulated frequency (AMF) was 50.0 Hz, spectrum was 10.0 Hz, and sweep frequency was set to sweeps.

**Figure 2:**
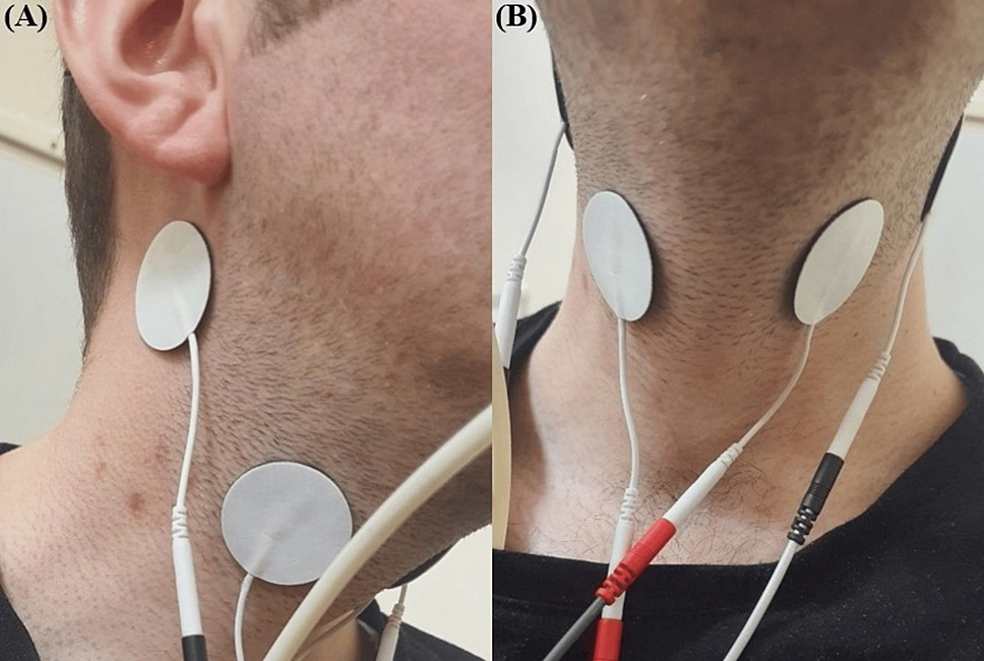
Adhesion points of the electrodes: (A) side view; (B) front view.

To perform the IFC, we used the BTL-4000 Smart® device (BTL Industries, Boston, Massachusetts), which is capable of generating an IFC with a pulse frequency of 50 Hz (Figure 1). As recommended in previous studies, this device was used to stimulate the nerves in the neck by placing two pairs of electrodes with different frequencies (2,000 and 2,050 Hz) [14-16,22] The anterior neck skin was cleaned, and then two independent pairs of electrodes were placed targeting the superior laryngeal nerve (Figure 2). One of the paired electrodes was placed in the submental region just below the angle of the mandible. The other co-electrode was placed transversely along the anterior ridge of the sternocleidomastoid muscle at the level of the laryngeal prominence. The stimulation intensity was set at 3 mA, which has been reported as the safe limit in previous studies [14-16,22]. Patients reported a slight vibration or tickling sensation at this intensity level but never complained of pain or tingling. No muscle contraction was observed at this stimulus intensity [14-16,22]. The 15-minute IFC stimulation treatment was applied once a day for a total of 15 sessions.

Written informed consent was obtained from all patients. All procedures in this case report were carried out in accordance with the latest version of the World Medical Association Declaration of Helsinki and the Good Clinical Practices Guidelines published by the Ministry of Health.

Case 1

**Figure 3:**
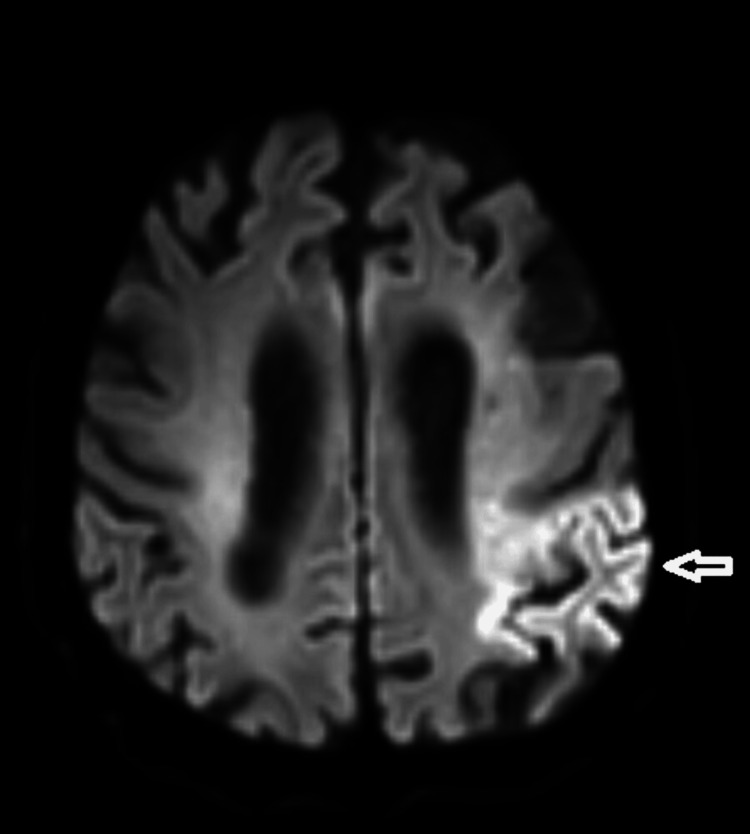
Diffusion-weighted imaging showed areas compatible with acute infarction in the left parietotemporal area (white arrow).

A 63-year-old man with a history of diabetes mellitus and hypertension was admitted to our clinic with dysphagia after an ischemic stroke four months ago. Magnetic resonance imaging (MRI) showed a left hemispheric infarct (Figure 3). He had a cough, a hoarse voice, and the need to clear the throat when taking thin liquids. He had not received any treatment before. Clinical examination revealed facial asymmetry, decreased oral motor skills, dysarthria, tongue deviation, unilateral decrease in pharyngeal reflex, and unilateral mild asymmetry in soft palate elevation. The patient had a hoarse cough, and cough strength and clarity were not complete. The FOIS score was 5. Based on these results, VFSS was scheduled for further evaluation. VFSS showed delayed swallowing reflex and premature spillage. Penetration was observed in semi-solids and liquids, and there was a contrast uptake in the laryngeal vestibule and in contact with the vocal folds in liquids. There was also dense residue in the vallecula and piriform sinuses after swallowing, especially with solids. The removal of the residue was insufficient. After 15 sessions of IFC treatment, the patient's FOIS score improved. Cough quality and severity improved. On the VFSS evaluation, the amount of residue decreased. The patient was able to clear the residue with dry swallowing after swallowing. The severity of laryngeal vestibular penetration decreased. The need for coughing and throat clearing decreased. The patient's PAS and ERS scores improved.

Case 2

**Figure 4:**
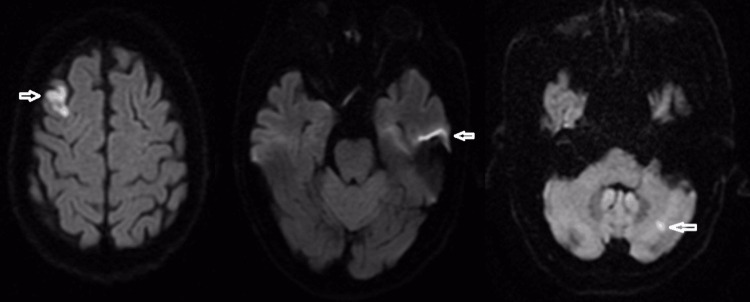
Diffusion-weighted imaging showed acute infarct areas in bilateral frontal lobes, predominantly on the right, and in the left half of the cerebellar hemisphere (white arrow).

A 31-year-old man with no comorbidities had an ischemic stroke secondary to aortic dissection three months ago and was admitted to our hospital for treatment. MRI scan showed bilateral areas of cortical infarction and left serebellar infarction (Figure 4). He had a history of intensive care. No tracheostomy was performed. He was fed by a nasogastric catheter. He had no previous treatment for dysphagia. Her oral intake was minimal and consisted of soft pudding-like foods. There was significant weight loss. He had unilateral vocal cord paralysis. Clinical examination revealed lip asymmetry, minimal hypernasality, mild dysarthria, dysphonia, and bilateral decrease in the pharyngeal reflex. Oral motor function was inadequate. Tongue movements were minimal, resulting in poor oral bolus preparation and transport. Cough strength and clarity were good. There was no tongue protrusion and the tongue could not reach the lip margin. He was tube-dependent and could take minimal solid and semi-solid food or liquid. The FOIS score was 2. Severe coughing, gagging, and choking were observed during feeding attempts. The passage of food was minimal, and the patient expectorated. VFSS performed for further evaluation showed delayed swallowing response and premature spillage. Aspiration was observed in semi-solids and liquids. In addition, there was intense residue in the vallecula and piriform sinuses, especially with solid foods. The anterior hyoid movement was insufficient. Laryngeal vestibule closure was impaired. There was inadequate inversion of the epiglottis. There was minimal passage of food through the upper esophageal sphincter. He was re-evaluated after 15 sessions of IFC treatment. VFSS showed no post-treatment aspiration and penetration of liquid food into the laryngeal vestibule and vocal folds. Epiglottis inversion and anterior hyoid movement increased. The amount of pharyngeal residue decreased. The PAS and ERS scores improved. Upper esophageal sphincter function improved and food passage increased. Oral intake increased and nasogastric catheter was removed. Tongue protrusion increased (11 mm from the upper lip margin). After treatment, compensatory maneuvers and dietary modification were recommended, and treatment was discontinued.

Case 3

**Figure 5:**
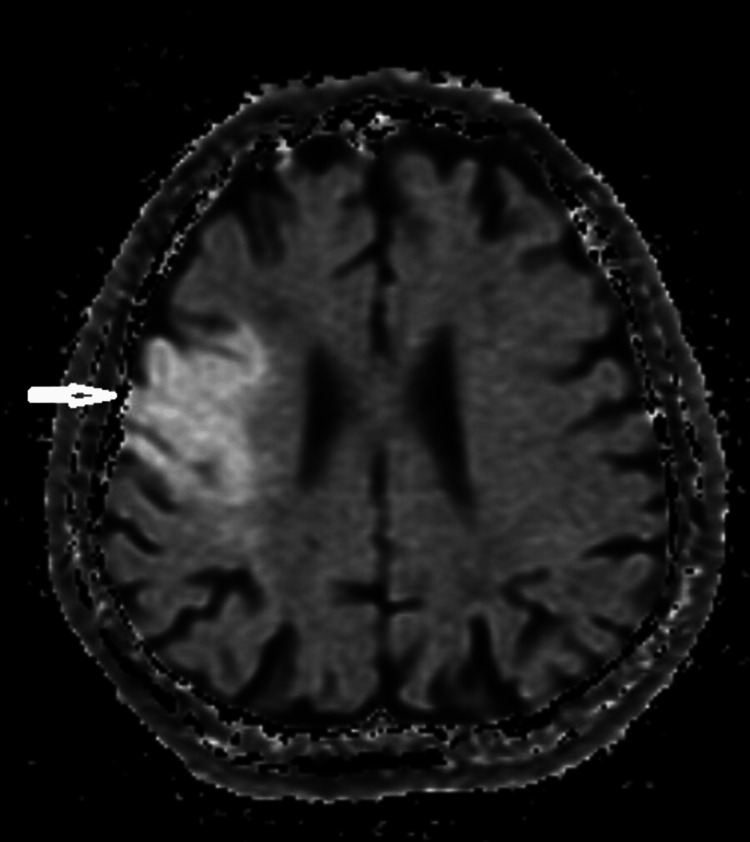
Diffusion-weighted imaging showed a large area of acute infarction in the anterior division of the cerebral artery irrigation area in the right midline (white arrow).

A 71-year-old woman patient with a history of hypertension and ischemic stroke 24 months ago was admitted to our clinic with dysphagia. MRI showed a right hemispheric infarct (Figure 5). A change in diet and consistency was previously recommended. She was fed semi-solid (e.g., puree and pudding) viscous foods. She did not practice postural maneuvers. She did not receive any rehabilitation program. The FOIS score was 5. Clinical evaluation revealed aphasia, facial asymmetry, and unilateral decrease in pharyngeal reflex and palatal reflex. Oral motor functions were reduced and tongue movements were minimal. There was no tongue protrusion. The cough reflex was reduced. Swallowing function was evaluated with food, and the patient required coughing and throat clearing during liquid intake. VFSS performed for further evaluation showed inadequate bolus preparation and transport, delayed swallowing response, and premature spillage. Aspiration was observed with liquids. In addition, there was residue in the vallecula and piriform sinuses, which was more pronounced with solid foods. After 15 sessions of IFC treatment, the patient's tongue movements improved, and tongue protrusion increased dramatically (13 mm). No liquid aspiration was observed on VFSS, with penetration of the laryngeal vestibule in contact with the vocal folds. The amount of residual in the vallecula and piriform sinuses decreased. Epiglottis inversion increased. The PAS and ERS scores improved.

The dysphagia assessment parameters of the patients before and after treatment are summarized in Table 1.

**Table 1:** Demographic characteristics and pre- and post-treatment parameters. FOIS, functional oral intake scale; PAS, penetration aspiration scale; ERS, Eisenhuber residue scale

	Case 1		Case 2		Case 3	
	Before treatment	After treatment	Before treatment	After treatment	Before treatment	After treatment
FOIS	5	6	2	5	5	6
PAS	5	2	7	5	6	4
ERS	3	2	3	2	2	1

## Discussion

IFC stimulation is a popular type of electrical stimulation used to stimulate muscles and is classified as intermediate frequency current. In IFC stimulation, two kilohertz alternating currents (ACs) of diagonally different frequencies are applied to produce a sinusoidal amplitude modulation at a frequency equal to the difference between the two ACs. The applied kilohertz frequency is called the carrier frequency, and the amplitude modulation frequency (=frequency difference) is called the beat frequency [23]. IFC reaches deeper tissue and is more comfortable for the patient than pulsed current. Intermediate-frequency currents allow the current to pass through the skin in a more tolerable manner [24]. Treatment of dysphagia with IFC is based on increasing oropharyngeal sensory input through afferent pathways using electrical stimulation [24]. Sensory stimulation therapy may be another potential strategy to treat dysphagia. This is because activation of peripheral sensory nerves in the larynx and pharynx may contribute to airway protection against aspiration [25]. It has also been shown that sensory stimulation strategies reduce the prevalence of VFS symptoms associated with impaired swallowing safety in patients with dysphagia [10].

To this end, some authors have reported that IFC stimulation at the sensory level can be applied during dysphagia rehabilitation [14-17]. It is hypothesized that IFC therapy helps to improve the swallowing reflex by increasing afferent stimulation, specifically by activating the superior laryngeal nerve, which triggers swallowing, without causing discomfort [26]. The internal branch of the superior laryngeal nerve, which innervates the epiglottis, laryngeal vestibule, and hypopharynx, is recognized as the most important sensory nerve that triggers cough and swallowing [27-29]. In another study aimed at investigating the underlying mechanism of IFC stimulation in the treatment of dysphagia, IFC was applied to deserebrated and paralyzed guinea pigs weakened by kainic acid injection into the nucleus tractus solitarius, and approximately half of the swallowing-related neurons responded to the stimulation. These data suggest that IFC stimulation applied to the neck activates the brainstem and/or cerebral cortex via sensory afferent nerves of the pharynx and larynx, thereby facilitating the swallowing reflex [11].

Few side effects of IFC have been described [14]. However, it may cause pain or discomfort in patients and includes possible risks of laryngospasm, arrhythmia, hypotension, glottic closure, and skin burns [30]. In a study aimed at investigating the safety of applying IFC stimulation to the neck, the carrier frequency and pulse frequency of the device were set to 2000 and 50 Hz, respectively, stimulation was performed at an intensity of 3 mA, and these parameters were found to be safe for sensory stimulation without visible muscle contraction. There is no clear value in the literature regarding current intensity. It is mostly described as the intensity at which the patient feels tingling. In the literature, this value is usually between 1 and 3 mA [13-16]. There is no standard value in this regard, and it is recommended that the stimulation intensity should not exceed 3 mA [22]. Given these data, we placed two pairs of electrodes diagonally under the mandible and on either side of the laryngeal prominence. We set the carrier frequency to 2000 Hz and the AMF to 50 Hz (beat frequency = 50 Hz). The stimulation intensity was set to 3 mA for 15 minutes in all three patients. Patients received a total of 15 sessions of IFC stimulation treatment.

There are no large-scale studies in the literature on the efficacy of IFC in the treatment of dysphagia. Studies have been conducted in small populations and outcome measures are limited. However, IFC treatment has been shown to significantly increase the number of swallows, improve the swallowing reflex, and lower the sensory threshold. This may reflect changes in plasticity in the cerebral cortex and may serve as an indicator of therapeutic efficacy [31,32]. In some studies aimed at demonstrating the efficacy of IFC in patients with dysphagia due to various etiologies, it has been shown that IFC therapy improves the cough reflex and feeding status, reduces aspiration, and regresses the PAS score. Based on these results, IFC stimulation may contribute to the prevention of aspiration pneumonia [13-15]. Other studies have reported that IFC stimulation can reduce dysphagia and chronic obstructive pulmonary disease (COPD) exacerbations in COPD patients with dysphagia [23,31]. It has also been reported that IFC stimulation can increase salivation in people with dry mouth without causing pain or physical distress [33].

There are also studies in the literature that have been conducted on healthy volunteers to measure the effectiveness of IFC. Furuta et al [16]. observed that IFC stimulation increased the number of swallows over a fixed period of time in healthy volunteers. Jungheim et al. [34] showed in a prospective study that the maximum base of tongue pressure increased by 8.4% after IFC stimulation. They recommended the use of IFC stimulation as an additional treatment tool, especially in patients with inadequate tongue base retraction. Izumi et al. [17] showed that IFC stimulation improved chewing ability in healthy volunteers. The evidence for the use of IFC in post-stroke dysphagia is still insufficient.

Specifically, there is only one article on the use of IFC stimulation in post-stroke dysphagia. This retrospective case-control study compared the efficacy of IFC therapy in addition to conventional therapy with conventional therapy, and the main outcome measure was the Food Intake Level Scale (FILS). The improvements in swallowing function and FILS score in the IFC group were significantly greater than those in the conventional treatment group. It was also observed that the average length of hospital stay was significantly shorter in the IFC group than in the conventional treatment group [35]. The limitations of this study are that although patients' swallowing function was evaluated by VFSS and endoscopy, the outcome measure was only FILS, which is an observational tool to assess nutritional status, and it was a retrospective study. All studies in the literature on the treatment of dysphagia with IFC stimulation have used Gentle Stim® (J Craft, Osaka, Japan), a portable device approved for use in Japan [13-18,22,23,26,31-33]. As in this study, we believe that an alternative device capable of providing IFC stimulation can also be used for treatment.

In our patients with poststroke dysphagia, regression of aspiration, reduction of residual bolus, improvement of tongue movements, improvement of bolus preparation and transport, and increase in cough strength and quality were generally observed. The patient who was fed by nasogastric catheter was free from nasogastric catheter after treatment and his feeding continued without tube dependence. Improvement in the FOIS, PAS, and ERS scores was observed in all cases. While tongue movements were minimal in two patients, a dramatic improvement in tongue movements, especially tongue protrusion, was observed after treatment. This was a remarkable finding. It has been reported in the literature that IFC stimulation can help to stimulate deeper muscles, such as genioglossus and hyoglossus, because it has a much shorter pulse duration than transcutaneous nerve stimulation and therefore can deliver maximum current to tissues with high tissue permeability [36,37]. In our case, we believe that tongue protrusion increased due to the effect of genioglossus stimulation with IFC. After a stroke, IFC stimulation can be used to improve tongue function.

## Conclusions

In these three case reports, the treatment of patients with post-stroke dysphagia using IFC stimulation is discussed in detail. The efficacy of IFC stimulation treatment was demonstrated by videofluoroscopic, objective scales. Therefore, we believe that IFC may be an alternative or complementary treatment modality to other treatment modalities for post-stroke dysphagia.

## References

[1] (2019). Global, regional, and national burden of neurological disorders, 1990-2016: a systematic analysis for the Global Burden of Disease Study 2016. Lancet Neurol.

[2] Umay E, Eyigor S, Ertekin C (2022). Best practice recommendations for stroke patients with dysphagia: a Delphi-based consensus study of experts in Turkey-part I: management, diagnosis, and follow-up. Dysphagia.

[3] Dziewas R, Michou E, Trapl-Grundschober M (2021). European Stroke Organisation and European Society for Swallowing Disorders guideline for the diagnosis and treatment of post-stroke dysphagia. Eur Stroke J.

[4] Blumenfeld L, Hahn Y, Lepage A, Leonard R, Belafsky PC (2006). Transcutaneous electrical stimulation versus traditional dysphagia therapy: a nonconcurrent cohort study. Otolaryngol Head Neck Surg.

[5] Martino R, Foley N, Bhogal S, Diamant N, Speechley M, Teasell R (2005). Dysphagia after stroke: incidence, diagnosis, and pulmonary complications. Stroke.

[6] Jung YJ, Kim HJ, Choi JB, Park JS, Hwang NK (2020). Effect of dysphagia rehabilitation using kinesiology taping on oropharyngeal muscle hypertrophy in post-stroke patients: a double blind randomized placebo-controlled trial. Healthcare (Basel).

[7] Shaw GY, Sechtem PR, Searl J, Keller K, Rawi TA, Dowdy E (2007). Transcutaneous neuromuscular electrical stimulation (VitalStim) curative therapy for severe dysphagia: myth or reality?. Ann Otol Rhinol Laryngol.

[8] Bergquist AJ, Clair JM, Lagerquist O, Mang CS, Okuma Y, Collins DF (2011). Neuromuscular electrical stimulation: implications of the electrically evoked sensory volley. Eur J Appl Physiol.

[9] Poorjavad M, Talebian Moghadam S, Nakhostin Ansari N, Daemi M (2014). Surface electrical stimulation for treating swallowing disorders after stroke: a review of the stimulation intensity levels and the electrode placements. Stroke Res Treat.

[10] Ortega O, Rofes L, Martin A, Arreola V, López I, Clavé P (2016). A comparative study between two sensory stimulation strategies after two weeks treatment on older patients with oropharyngeal dysphagia. Dysphagia.

[11] Umezaki T, Sugiyama Y, Fuse S, Mukudai S, Hirano S (2018). Supportive effect of interferential current stimulation on susceptibility of swallowing in guinea pigs. Exp Brain Res.

[12] Aviv JE, Martin JH, Sacco RL, Zagar D, Diamond B, Keen MS, Blitzer A (1996). Supraglottic and pharyngeal sensory abnormalities in stroke patients with dysphagia. Ann Otol Rhinol Laryngol.

[13] Funato M, Maruta K, Yano M (2023). Efficacy of interferential current transcutaneous electrical sensory stimulation through the neck skin for treating dysphagia in children with disabilities: a case series. SAGE Open Med Case Rep.

[14] Maeda K, Koga T, Akagi J (2017). Interferential current sensory stimulation, through the neck skin, improves airway defense and oral nutrition intake in patients with dysphagia: a double-blind randomized controlled trial. Clin Interv Aging.

[15] Hara Y, Nakane A, Tohara H (2020). Cervical interferential current transcutaneous electrical sensory stimulation for patients with dysphagia and dementia in nursing homes. Clin Interv Aging.

[16] Furuta T, Takemura M, Tsujita J, Oku Y (2012). Interferential electric stimulation applied to the neck increases swallowing frequency. Dysphagia.

[17] Iizumi Y, Ihara Y, Koike J, Takahashi K (2023). Effects of interferential current electrical stimulation (IFCS) on mastication and swallowing function in healthy young adults: a preliminary study. Clin Exp Dent Res.

[18] Crary MA, Mann GD, Groher ME (2005). Initial psychometric assessment of a functional oral intake scale for dysphagia in stroke patients. Arch Phys Med Rehabil.

[19] Logemann J, Pauloski B, Rademaker A, Kahrilas P (2002). Oropharyngeal swallow in younger and older women: videofluoroscopic analysis. J Speech Lang Hear Res.

[20] Rosenbek JC, Robbins JA, Roecker EB, Coyle JL, Wood JL (1996). A penetration-aspiration scale. Dysphagia.

[21] Eisenhuber E, Schima W, Schober E, Pokieser P, Stadler A, Scharitzer M, Oschatz E (2002). Videofluoroscopic assessment of patients with dysphagia: pharyngeal retention is a predictive factor for aspiration. AJR Am J Roentgenol.

[22] Nagami S, Maeda K, Fukunaga S, Ikeno M, Oku Y (2019). Safety of transcutaneous electrical sensory stimulation of the neck in terms of vital parameters in dysphagia rehabilitation. Sci Rep.

[23] Oku Y (2023). Swallowing disorder - a possible therapeutic target for preventing COPD exacerbations. Respir Physiol Neurobiol.

[24] Petrofsky J (2008). The effect of the subcutaneous fat on the transfer of current through skin and into muscle. Med Eng Phys.

[25] Tsujimura T, Udemgba C, Inoue M, Canning BJ (2013). Laryngeal and tracheal afferent nerve stimulation evokes swallowing in anaesthetized guinea pigs. J Physiol.

[26] Sugishita S, Imai T, Fukunaga S, Matsui T (2018). Delayed swallowing reflex improved by interferential current stimulation in combination with direct therapy [Article in Japanese]. Japan J Dysphagia Rehabilitation.

[27] Oku Y, Tanaka I, Ezure K (1994). Activity of bulbar respiratory neurons during fictive coughing and swallowing in the decerebrate cat. J Physiol.

[28] DO RW (1951). Influence of stimulus pattern on reflex deglutition. Am J Physiol.

[29] Hiroto I, Toyozumi Y, Yatake Y (1968). Comparative anatomy of the laryngeal nerves of mammals [Article in Japanese]. Nihon Jibiinkoka Gakkai Kaiho.

[30] Lim KB, Lee HJ, Lim SS, Choi YI (2009). Neuromuscular electrical and thermal-tactile stimulation for dysphagia caused by stroke: a randomized controlled trial. J Rehabil Med.

[31] Yoshimatsu Y, Tobino K, Nishizawa S, Yoshimine K, Oku Y (2022). Interferential current stimulation for swallowing disorders in chronic obstructive pulmonary disease: a preliminary study. Prog Rehabil Med.

[32] Oku Y, Sugishita S, Imai T (2015). Effects of short term interferential current stimulation on swallowing reflex in dysphagic patients. Int J Speech Lang Pathol Audiol.

[33] Hasegawa Y, Sugahara K, Sano S, Sakuramoto A, Kishimoto H, Oku Y (2016). Enhanced salivary secretion by interferential current stimulation in subjects with dry mouth: a pilot study-reply. Oral Surg Oral Med Oral Pathol Oral Radiol.

[34] Jungheim M, Schubert C, Miller S, Ptok M (2017). Swallowing function after continuous neuromuscular electrical stimulation of the submandibular region evaluated by high-resolution manometry. Dysphagia.

[35] Waza M, Hayashi Y, Sakurai T (2017). Efficacy of interferential currents stimulation on post-stroke dysphagia: a case control study. J Neurol Sci.

[36] Cho SH, Kim SC (2020). Changes in electroencephalography by modulation of interferential current stimulation. Appl Sci.

[37] Barikroo A (2020). Transcutaneous electrical stimulation and dysphagia rehabilitation: a narrative review. Rehabil Res Pract.

